# Rheumatisms

**Published:** 1905-12-02

**Authors:** 


### RHEUMATISMS.

There are few terms in medical dialectics more heavily burdened with responsibility than the word "rheumatism"; and it must be confessed that half its burden at least has to be credited to loose talking by the profession. The public grows daily more curious in medical matters, and demands with ever-increasing insistence a dogmatic label for the symptom or combination of symptoms which vexes it at the moment. It is little matter for wonderment, therefore, that the severely heckled and harassed doctor should buy immunity from questions at the price of loose and misleading generalisations. One of these days the word "rheumatism" will be confined by general consent (among the profession at least) to the lesions of the micrococcus of rheumatism. This happy consummation is still probably a long way off, for there is much to be done in the way of methodical analysis of the "rheumatisms," and conclusions in the matter have to be reached along the difficult path of induction, since ocular demonstration of the essentials of the various lesions is but seldom practicable. But such a paper as that of Dr. Luff [1] marks a distinct advance, for it attempts an analysis of the "rheumatisms" which are neither rheumatism in its true application nor osteo-arthritis.

The pathology of the lesions with which this author deals is in his opinion essentially an inflammatory hyperplasia of the white fibrous tissue in various parts of the body, a "fibrositis." The articular structures proper—that is to say, synovial membrane, bone and cartilage—are not primarily affected; the stress of the lesion falls upon the aponeuroses and insertions of the muscles, the fasciae, the ligaments of the joints, and the periosteum. This inflammatory hyperplasia occurs in patches; it forms well-defined indurations situated in any of the structures above-mentioned. The pain associated with these conditions depends upon the tension of muscular movement in the inflamed areas. In the case of muscle the pain is conveyed by the muscle-spindles, which are the sole sensory structures in muscle. Involvement of a nerve by such a fibrous induration may lead to a diffusion of the pain over an extensive area.

The various forms of "fibrositis" which masquerade under the guise of rheumatism include the following in chief:

1. *Muscular rheumatism.*—This is always a fibrositis, and it may affect any area of the body. The pain is at first generally dull, but becomes much aggravated by movement or the onset of damp weather. The pain is usually most pronounced when the patient becomes warm in bed, and is also severe when he rises from bed in the morning.

2. *Lumbago.*—This is a typical form of fibrositis. It may be an affection of the lumbar muscles, but more commonly begins, according to Dr. Luff, as a localised affection of the muscular insertions situated in the neighbourhood of the sacro-iliac articulations. This view is based upon the frequency with which, in the course of examining a patient with lumbago, pressure upon the muscles of the loin has been found to be unattended by pain, while pressure upon the sacro-iliac joints has been greatly complained of.

3. *Rheumatic neuralgia.*—This is a fibrositis of nerve-sheaths, and is a frequent cause of sciatica. The affected nerve is painful when stretched or pressed; the associated symptoms are numbness, pins-and-needles, and pain.

4. *Dupuytren's contraction.*—A fibrositis caused by an habitual, postural use of the hand. Though ascribed to gout, this condition is not confined to gouty subjects. Nor has the author succeeded in demonstrating gouty deposits in the thickened tissues.

The etiology of fibrositis embraces the following possibilities:

(*a*). Cold, damp, and wet. Any combination of these appears to be an efficient cause in a predisposed subject. When the ailment is established, the exacerbations of the pain correspond, not necessarily with rainy weather, but with the mere fall of barometric pressure.

(*b*). Extremes of temperature. Sudden and extreme variations in temperature will produce fibrositis. A severe example of the disease, quoted by Dr. Luff, occurred in a fireman who, after working in the stokehold in the great heat of the Red Sea, went direct to the refrigerating chamber to cool himself. The result was an inflammation of the fibrous tissues throughout the body which completely prostrated him, and from which he recovered only after a tedious and prolonged convalescence.

(*c*). Injuries are capable of setting up a local fibrositis; golf is credited with certain specially localised lesions, analogues, we suppose, of the "tennis-elbow."

(*d*). Toxæmias of various orders cause temporary fibrositis; for instance, tonsillitis, in fluenza, and too free an indulgence in good living are well recognised causes of muscular pains.

Touching treatment, Dr. Luff recommends for internal administration aspirin as an analgesic, and iodide of potash as a curative agent; the latter in doses of ten or twelve grains, combined with nux vomica or the compound glycerophosphate syrup. For external application a paint composed of equal parts of chloral hydrate, menthol, and camphor (which liquefy when they are mixed) should be painted upon the affected surface, and then gently rubbed in with the fingers. A second useful application is the tincture of iodine as a paint, followed by a hot poultice or fomentation which vaporises the iodine and has a well-marked analgesic effect.

Heat, massage, electricity, and exercises all have their value in the treatment of fibrositis; but dogmatic restrictions in the matter of dietary are repudiated by the author. The following paragraph is a welcome antidote to much latter-day humbug. "No special dieting is required in these affections. Moderation should be the keynote of all prone to the various forms of fibrositis, and especially should they avoid foods which their experience has taught them to be apt to set up gastro-intestinal fermentation. I am, however, absolutely opposed to the indiscriminate prohibition of such articles as red meats, sugar, jams, bread, fruit, etc., in the cases of sufferers from the various forms of fibrositis. All these articles of food, if they do not produce indigestion, are perfectly wholesome. It has become of late years a fashionable craze to attribute many of these forms of 'chronic rheumatism' to uric acid. I most emphatically declare that it has no part in the production of any of the morbid conditions dealt with in this lecture. Uric acid is a harmless by-product of the human economy which has been most shamefully exploited as a dangerous poison."

[1] Clin. Jour., October 11.

